# Behavioural discrimination of noxious stimuli in infants is dependent on brain maturation

**DOI:** 10.1097/j.pain.0000000000001425

**Published:** 2018-12-17

**Authors:** Gabrielle Green, Caroline Hartley, Amy Hoskin, Eugene Duff, Adam Shriver, Dominic Wilkinson, Eleri Adams, Richard Rogers, Fiona Moultrie, Rebeccah Slater

**Affiliations:** a Department of Paediatrics, University of Oxford, Oxford, United Kingdom; b The Oxford Uehiro Centre for Practical Ethics, University of Oxford, Oxford, United Kingdom; c Nuffield Department of Anaesthesia, John Radcliffe Hospital, Oxford, United Kingdom

**Keywords:** Facial expression, Behaviour, EEG, Preterm, Noxious, Innocuous, Evoked response, Brain activity

## Abstract

Changes in facial expression are an essential form of social communication and in nonverbal infants are often used to alert care providers to pain-related distress. However, studies of early human brain development suggest that premature infants aged less than 34 weeks' gestation do not display discriminative brain activity patterns to equally salient noxious and innocuous events. Here we examine the development of facial expression in 105 infants, aged between 28 and 42 weeks' gestation. We show that the presence of facial expression change after noxious and innocuous stimulation is age-dependent and that discriminative facial expressions emerge from approximately 33 weeks' gestation. In a subset of 49 infants, we also recorded EEG brain activity and demonstrated that the temporal emergence of facial discrimination mirrors the developmental profile of the brain's ability to generate discriminative responses. Furthermore, within individual infants, the ability to display discriminative facial expressions is significantly related to brain response maturity. These data demonstrate that the emergence of behavioural discrimination in early human life corresponds to our brain's ability to discriminate noxious and innocuous events and raises fundamental questions as to how best to interpret infant behaviours when measuring and treating pain in premature infants.

## Abstract 2

Supplemental Digital Content is Available in the Text.

Facial expression discrimination between noxious and innocuous stimuli in preterm infants emerges at approximately 33 weeks' gestation and relates to evoked brain activity maturation.

## 1. Introduction

Facial expressions in infants facilitate social interaction^16^ and provide a mechanism by which infants can alert care providers to their pain or distress.^33^ This immature form of social communication elicits intervention and ultimately protects infants from aversive situations. Hospitalised infants regularly undergo painful medical procedures,^7^ and facial expressions form the cornerstone of infant pain assessment.^28^ Pain perception and observed pain-related facial expressions have been related in adults.^27^ However, this may not be the case in premature infants due to the immaturity of the developing nervous system.^11,19^ In the most premature infants, facial expressions can be observed after procedural touch stimulation,^15^ and it is unclear whether premature infants display discriminative behaviours after salient noxious and innocuous events.^1^ To improve the measurement of pain in nonverbal hospitalised infants, we need a greater understanding of how pain-related facial expressions emerge and develop in early life.

Discriminative patterns of evoked brain activity emerge across the preterm period. In younger gestation infants, nondiscriminative activity known as a delta brush, which may have origins in the insula,^3^ is evoked by both innocuous and noxious inputs,^12^ as well as auditory and visual stimuli.^9,10^ By contrast, older infants from approximately 34 to 35 weeks' gestation generate noxious-evoked brain activity with specific morphology that is not evoked by visual, tactile, or auditory inputs.^12,18^ We hypothesised that, consistent with the maturation of discriminative patterns of brain activity, changes in facial expression discriminating between noxious and innocuous stimulation will emerge during the preterm period. We examined the development of facial expressions in infants aged between 28 and 41 weeks' gestation, investigating their responses to acute procedural noxious and innocuous stimulation. In a subset of infants, we recorded their brain activity responses using EEG to determine how facial expression relates to the maturation of evoked brain activity.

## 2. Methods

### 2.1. Participants

Between April 2012 and May 2017, a total of 122 infants were recruited from the Newborn Care Unit and Maternity wards of the John Radcliffe Hospital, Oxford University Hospital NHS Foundation Trust, Oxford, United Kingdom. Infants were born between 23 and 42 weeks' gestation and were between 28 and 42 weeks' gestational age at the time of study. One hundred and five infants were included in an analysis of facial expressions and 49 infants were included in an analysis of evoked brain activity across the preterm period. A sample of 18 term infants were used to characterise the non–modality-specific pattern of EEG activity recorded in response to both noxious and tactile stimulation, and to derive an EEG template of this sensory-evoked activity (see supplementary Figure 1 for further detail, available at http://links.lww.com/PAIN/A686).

Infants were not included in the study if they had documented neurological malformations, intraventricular hemorrage (IVH) greater than grade 2, or a history of maternal substance abuse. At the time of study, all infants were clinically stable and not requiring invasive ventilation. None of the infants had received analgesics or sedatives in the preceding 72 hours. Infant demographics were recorded at the time of study from the clinical notes and are described in Table 1. To estimate cumulative pain exposure, we retrospectively reviewed the number of oropharyngeal aspirations, tracheal aspirations, and tissue-damaging procedures performed for blood taking (which included heel lances, venipuncture, and intravenous cannulation) that were documented in the electronic and paper clinical records between birth and the time of study. These procedures were selected as they are among the top 6 most common painful procedures experienced by infants in neonatal intensive care.^7^ They have been used in previous studies^19^ and are clinically well documented, facilitating retrospective review. We did not ascertain the number of attempts for each procedure (as this information is not available retrospectively); however, the estimate produced by this method provides a consistent way of comparing infant pain exposure across the study population.

**Table 1:**
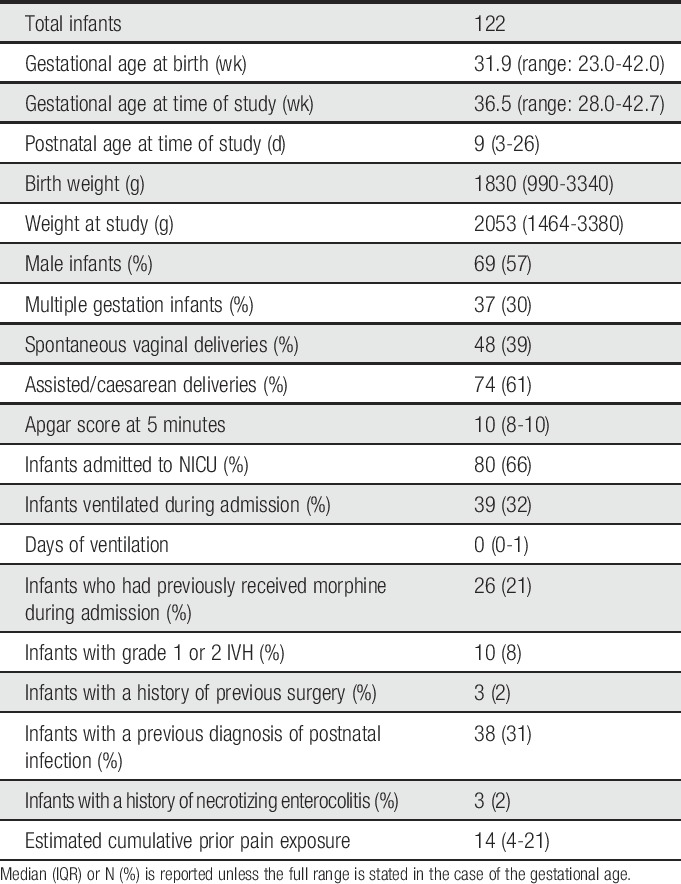
Infant demographics.

Ethical approval was obtained from the National Research Ethics Service (references: 12/SC/0447 and 11/LO/0350) and written informed parental consent was gained before each participant was studied. The study was conducted in accordance with the standards set by the Declaration of Helsinki and Good Clinical Practice guidelines.

### 2.2. Experimental procedures

#### 2.2.1. Heel lancing and control heel lance

All heel lances were performed when clinically required as part of the infant's medical care. Heel lances were not performed solely for the purpose of the study. Care was taken to ensure that the infants were given appropriate comfort, such as swaddling or containment holding, according to gestational age and parental preference. When the infants were swaddled, they were laid on a cotton cloth with their arms crossed over their chest in a relaxed position. The ends of the cloth were then crossed over the infant's body and arms, and tucked beneath the opposing side. The swaddling cloth restricted gross body movements and held the infant securely and comfortingly in a flexed position, without covering their feet to allow access for blood taking. The choice of foot for heel lancing was based on clinical judgment and not controlled during the experiment. Heel lances were performed on the medial or lateral plantar surface of the heel. In term infants, a BD Microtainer Quikheel Infant Lancet (Becton, Dickinson and Company, Franklin Lakes, NJ) with a penetration depth of 1.0 mm was used and, in preterm infants, a BD Quikheel Preemie Lancet with a penetration depth of 0.85 mm was used. After the lance, the foot was not squeezed for 30 seconds to ensure the observed response was only to the stimulus applied. Before the lance, a control lance was performed whereby the lancet was rotated by 90° and held against the infant's foot so that when the lance was released, there was no contact with the infant's heel. A video camera was used to record facial expressions throughout the procedure for post hoc analysis. The timings of the lance and control lance were marked on the video using an LED, which flashed when the person performing the lance pressed a foot pedal at the point of stimulation.

#### 2.2.2. EEG recordings

In 49 infants, brain activity was recorded during the stimuli using EEG. EEG from DC to 400 Hz was acquired with a SynAmps RT 64-channel EEG/EP system (Compumedics Neuroscan). Activity was recorded with a sampling rate of 2000 Hz using CURRYscan7 neuroimaging suite (Compumedics Neuroscan). To optimise contact with the scalp, the skin was gently rubbed with EEG preparation gel (NuPrep gel; D.O. Weaver and Co, Aurora, CO) before electrode placement and application of EEG conductive paste (Elefix EEG paste; Nihon Kohden). EEG was recorded at the Cz, CPz, C3, C4, FCz, Oz, T3, and T4 electrode sites, with the reference electrode at Fz and a ground electrode on the forehead. In 11 infants, EEG was recorded at Cz, CPz, C3, and C4 only. Stimuli were time locked to EEG recordings using an accelerometer as previously described.^46^

### 2.3. Analysis

#### 2.3.1. Facial expression scores

Analysis of the facial expression was undertaken after acquisition by research assistants, who had been trained in Premature Infant Pain Profile-Revised (PIPP-R) scoring and who had achieved high levels of interrater and intrarater reliability before the study analysis. The baseline behavioural state was scored in the 15 seconds preceding the control lance and again in the 15 seconds preceding the heel lance. A score between 0 and 3 was given according to whether the infant was active and awake, quiet and awake, active and asleep, or quiet and asleep, respectively, as per the Premature Infant Pain Profile—Revised (PIPP-R).^42^

The presence of 3 facial expressions (brow bulge, eye squeeze, and nasolabial furrow) was assessed in the 30 seconds after both the control lance and heel lance. The duration of facial expression activity was defined as the duration during which any of the 3 facial expressions were observed, whether together or individually, to a maximum of 30 seconds. The duration of facial expressions was timed using a stopwatch. If the infant stopped displaying a facial expression, the timer was paused, and if any expression was seen again during the 30-second period, the timer was restarted to give a cumulative time. An infant was considered to have a facial expression response to a stimulus if the infant displayed any response during the 30-second period (ie, if the duration of the response was greater than 0).

Each facial expression was also taken individually to calculate a facial expression score using the facial component of the PIPP/PIPP-R.^41,42^ A score between 0 and 3 for each facial expression was given accordingly, giving a total score between 0 and 9. Observers were blinded to the stimulus type when scoring the videos. Interrater and intrarater reliability were calculated using intraclass correlation of the PIPP scores. This was performed for 20% of the videos that were rescored by the first observer, and 35% of the videos that were rescored by a second independent observer. Videos were selected at random for rescoring. The intrarater reliability was 0.95 and the interrater reliability was 0.96.

#### 2.3.2. EEG analysis

EEG activity was filtered from 0.5 to 70 Hz with a notch filter at 50 Hz. The data were epoched from 4 seconds before and after the stimulus, and baseline corrected to the prestimulus mean. Individual EEG channels contaminated with artefacts, such as movement artefact, were removed from the analysis.

Noxious-specific brain activity was identified from recordings at the Cz electrode site using a previously described template of noxious-evoked brain activity.^18^ Individual EEG traces were first Woody filtered by a maximum jitter of ±50 ms to achieve maximal correlation of the data and the template. The template was then projected onto the data in the time window from 400 to 700 ms after the stimulus to obtain the magnitude of the noxious-evoked brain activity, as previously described.^18^ The same process was repeated with background EEG recordings–where the infant's foot was gently held but no stimuli were applied. This gave a distribution of the magnitude of the brain activity within the background data, and a threshold of 80% of this background distribution was set as the threshold for noxious-specific brain activity (a value of 0.34). Thus, if the response to the heel lance was above this threshold in any given infant, noxious-specific brain activity was said to have occurred.

In term infants, both tactile and noxious stimuli have been shown to evoke an earlier potential approximately 250 ms after the stimulus.^12,38^ An independent sample of 18 term infants (which were the same group of infants used to derive the template of noxious-evoked brain activity in a previous publication^18^) was used to characterise this response at the Cz electrode. Activity in the background period and in response to a control lance, heel lance, experimental noxious stimulation (128 mN PinPrick; MRC systems), and experimental tactile stimulation (modified tendon hammer) was epoched from 1 second before to 1.5 seconds after the stimulus, filtered from 0.5 to 8 Hz to allow the response to be characterised without being affected by artefacts, and baseline corrected to the prestimulus mean (for further details of the stimuli and experimental design, see Study 1 from Hartley et al.^18^). The data were first Woody filtered to the average response in the time window 100 to 300 ms after the stimulus, with a maximum jitter of ±50 ms to account for variation in latency with individual infants. Principal Component Analysis was then conducted on the data in the same time window. The first 2 principal components accounted for 95% of the variance and were therefore the only components considered. The weights of the first component were significantly higher in response to the tactile and noxious stimuli compared with background activity, indicating that the component was related to the stimulation (*P* < 0.05, linear mixed-effects analysis with Tukey post hoc comparisons). By contrast, the weights of the second principal component were not significantly different between modalities (*P* = 0.23, linear mixed-effects model); so, the first principal component was selected as the template of the sensory-evoked potential. This template was then projected onto individual trials in the study data set at the Cz electrode using singular value decomposition to calculate the magnitude of the sensory-evoked potential within each individual trial (data were first Woody filtered to the template in the time window 100-300 ms after the stimulus).^12,18^ Similarly, to the noxious-specific brain activity, this process was also performed with the background brain activity to obtain a distribution of the background data. A threshold of 80% of this background distribution (a value of 0.50) was used to define the occurrence of the sensory-evoked potential within individual trials.

The presence of delta brushes was investigated using a previously described burst detection method based on the co-occurrence of slow waves (0.5-2 Hz) and higher frequency activity (8-22 Hz).^17^ Delta brushes were said to occur in response to stimulation if they occurred at any electrode site and the start of the nested higher-frequency activity was within 2 seconds of the point of stimulation (this point was taken because the algorithm detects a delta brush as the co-occurrence of the slow- and high-frequency activity and so defines the start as the start of the high-frequency activity, not the slow-frequency activity that will begin earlier).^12^

### 2.4. Statistical analysis

The MATLAB programming environment was used to conduct all statistical analyses. Generalised linear regressions with logit link functions were used to describe the proportion of infants with facial expression responses and different patterns of brain activity, across gestational age. For this, proportions were first calculated in 2-week intervals (starting at 28-30 weeks) with intervals overlapping by 1 week. To ensure that the number of infants was well distributed across the age range, a minimum of 12 infants were included in each 2-week interval. A generalised linear regression was also used to assess the proportion of infants with discriminative facial expressions according to brain response maturity. Comparison of the duration of facial expression response to the innocuous and noxious stimulation was performed using Wilcoxon signed rank tests.

Gaussian process modelling was used to model the development of response probabilities to innocuous and noxious stimuli with age, to assess the point of divergence of facial expression discrimination.^35^ For each of the 2 stimuli independently, the probability of infants responding to the stimulus was modelled across age using a squared exponential with automatic relevance determination covariance function, with a characteristic length scale of 3.5 weeks and an SD of 1. The Gaussian process models smooth changes of the likelihood of the infants to respond to the stimulus with age. An error function likelihood was used, and inference was achieved using expectation propagation.

Once the models were fitted, the distribution of modelled response likelihoods at any given age were compared and used to assess the divergence of response probabilities for the 2 stimuli. Using a Gaussian approximation to the standard error of response likelihoods, approximate *P*-values were generated to quantify the magnitude of this difference at a particular age. Note that these *P*-values do not reflect independent statistical tests, but indicate, given the model, the expected proportion of experiments where the difference in response likelihoods across stimuli matches that observed.

## 3. Results

We recorded the facial expressions of 105 clinically stable infants, aged between 28 and 41 weeks' gestation, during a clinically required noxious heel lance (for blood testing) and an innocuous control procedure (control heel lance). The presence and duration of 3 facial expressions—brow bulge, nasolabial furrow, and eye squeeze, as described in the Premature Infant Pain Profile^41,42^—were assessed in the 30 seconds after the stimuli. Infant demographics are shown in Table 1.

**Figure 1.**
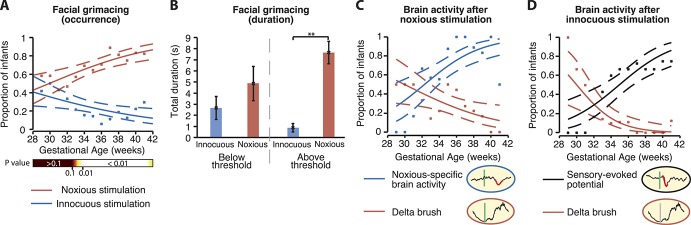
Facial expression and brain activity discrimination emerges with increasing gestational age. (A) The proportion of infants who displayed facial expressions after noxious stimulation (red) and innocuous stimulation (blue) according to gestational age. Proportions were calculated in 2-week intervals, with intervals overlapping by 1 week. The fit from a generalised linear model (solid lines) and 90% confidence intervals (dashed lines) are overlaid. The bottom colour bar indicates the *P*-values for the difference in proportions between the 2 groups calculated using Gaussian process modelling. The proportion of infants younger than 32 weeks' gestation who responded to the noxious stimuli shows substantial overlap with the proportion who responded to innocuous stimulation (*P* > 0.1), whereas in infants older than 33.9 weeks' gestation, infants are significantly more likely to display facial expressions after noxious stimulation compared with innocuous stimulation (*P* < 0.01, Gaussian process model). (B) Infants younger than 32 weeks also had no significant difference in the duration of their facial expression after noxious or innocuous stimulation, whereas facial expression responses of infants older than 33.9 weeks' gestation were significantly longer in duration after the noxious stimulation compared with the innocuous stimulation (**P < 0.001). (C) The proportion of infants who exhibited noxious-specific brain activity (blue) or non–modality-specific delta brush responses (red) to the noxious stimulation. (D) The proportion of infants who exhibited a sensory-evoked potential (black) and delta brushes (red) after innocuous stimulation.

Overall, 24% of infants displayed a facial expression response after the innocuous stimulus and 69% displayed a facial expression response to noxious stimulation. However, the likelihood that facial responses were observed was age-dependent (Fig. 1A). The proportion of infants who displayed a noxious-evoked facial expression significantly increased with gestational age (*P* = 0.0005, generalised linear regression, coefficient β = 0.16, 95% confidence interval [CI]: 0.07-0.24, Fig. 1A), whereas the proportion of infants who displayed a facial expression to the innocuous stimulation significantly decreased with gestational age (*P* = 0.014, β = −0.11, 95% CI: −0.21 to −0.02, Fig. 1A).

Using Gaussian process modelling,^35^ a supervised machine learning method that can model changes in the probabilistic state of populations, we identified that the responses diverge from 33.0 weeks' gestation, at which point there is a 95% probability that the average proportion of infants responding to noxious stimuli is greater than the average proportion of infants responding to the innocuous stimuli (Fig. 1A). By 33.9 weeks' gestation, this probability increases to 99%. Before 32 weeks' gestation, there is a substantial overlap in the distributions of infants responding to the noxious and innocuous stimuli (*P* > 0.1).

The duration of the facial expressions also demonstrated a similar developmental profile. In infants younger than 32 weeks' gestation, there was no significant difference in the duration of the evoked facial expression after either stimuli (median difference: 2.35, 95% CI: −1.10-8.00, *P* = 0.16, N = 26, Wilcoxon signed rank test, Fig. 1B). By contrast, in infants older than 33.9 weeks' gestation, the duration of the noxious-evoked facial activity was significantly greater than the duration of facial activity evoked by the innocuous stimulation (median difference: 7.85, 95% CI: 5.75-11.10, *P* < 0.001, N = 65, Wilcoxon signed rank test, Fig. 1B). This suggests that age-dependent maturation of discriminative facial expressions may permit older infants to better communicate their pain experience.

The age of emergence of the development of discriminative modality-specific brain activity patterns has been relatively well characterised^12,19^ and seems consistent with the behavioural maturation observed here. We recorded brain activity responses to the noxious and innocuous stimuli in a subset of 49 infants using EEG, and assessed the presence of (1) noxious-specific brain activity, (2) sensory-evoked brain activity (an evoked potential elicited by either noxious or innocuous stimulation),^38^ and (3) delta brushes (see Methods). Consistent with previous observations in premature infants,^9,10,12^ we observed that younger, more premature, infants are more likely to display delta brushes and that older infants more likely display noxious-specific activity after the heel lance. Overall, 67% of infants in this population exhibited noxious-specific brain activity after the heel lance, and the proportion significantly increased with gestational age (*P* = 0.0005, generalised linear regression, β = 0.30, 95% CI: 0.14-0.48, Fig. 1C). Only 1 of the 8 infants (12.5%) who were younger than 32 weeks' gestation had noxious-specific brain activity after the heel lance, compared with 27 of 33 (82%) infants older than 33.9 weeks' gestation. In a recent article, we did not report any noxious-specific brain activity in infants younger than 32 weeks' gestation,^19^ but this is likely to reflect the small sample size, and the results here demonstrate that although it is possible to observe noxious-specific brain activity at this age, consistent with previous reports,^12^ this occurs with increasingly lower frequency in younger gestational infants.^44^ In the most premature infants, delta brush activity was more likely to be evoked in response to both the noxious and innocuous stimuli, rather than the more mature modality-specific activity patterns (Figs. 1C, D). These data confirm that the emergence of modality-specific brain activity and discriminative facial expressions occur over the same developmental time window, at approximately 33 weeks' gestation.

**Figure 2.**
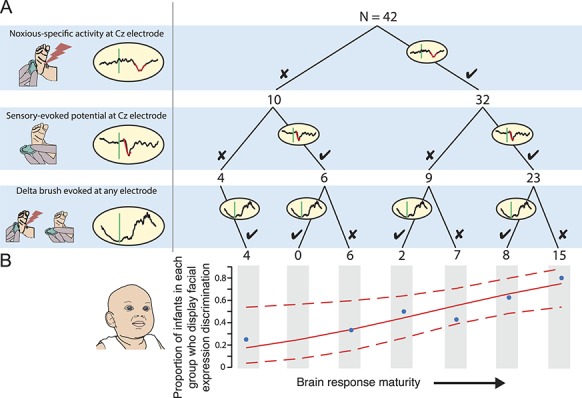
Facial expression discrimination depends on brain response maturity. (A) A decision tree was used to classify infants according to brain response maturity based on their responses to both noxious and innocuous stimuli. Noxious-specific brain activity and sensory-evoked potentials are seen in more mature, older infants. By contrast, delta brush responses are more immature. Therefore, infants classified on the right of the tree have more mature responses compared with infants on the left. The first split of the tree classifies infants dependent on the presence of noxious-specific brain activity; the second split classifies infants dependent on the presence of a sensory-evoked potential; and the final split considers whether delta brush activity was recorded at any electrode. Crosses indicate that a particular type of brain activity was not present, whereas ticks indicate that this type of brain activity was present. The number of infants classified at each split is indicated at the bottom of the branches. (B) Brain response maturity classification determined from the decision tree is plotted against the proportion of infants in each group that display facial expression discrimination (a response to the noxious stimulus but not the innocuous stimulus). The fit from a generalised linear model (solid lines) and 90% confidence intervals (dashed lines) are overlaid.

In the subset of 46 infants who had both facial expression and brain activity recorded, 44 infants had facial expression and EEG activity recorded in response to both the control heel lance and the heel lance without artefact. We investigated whether the infants' ability to display behavioural discrimination was dependent on the maturity of their brain activity responses. We used a decision tree to categorise infants based on the maturity of their brain activity (Fig. 2A). We defined infants with the least mature brain responses as those with only evoked delta brushes, and infants with the most mature responses as those with both noxious-specific brain activity in response to the heel lance and a sensory-evoked potential in response to either the noxious or innocuous stimulation. Two infants who did not display any type of evoked response, and therefore whose response maturity could not be classified, were excluded from the analysis, leaving 42 infants in the decision tree. The proportion of infants who displayed discriminative facial expressions (defined as a behavioural response to the noxious but not the innocuous stimulus) in each group was calculated.

The infants' ability to display discriminative facial expressions significantly increased with increasing maturity of the infants' brain responses (*P* = 0.016, generalised linear regression, β = 0.49, 95% CI: 0.11-0.93, Fig. 2B). Seventeen of the 23 infants (74%) with mature brain activity patterns (noxious-specific and sensory-evoked brain activity) displayed discriminative facial expressions. By contrast, in infants with immature brain activity (ie, where noxious-specific brain activity or sensory-evoked brain activity were not generated), only 7 of the 19 infants (37%) displayed discriminative facial expression responses. This demonstrates that an infant's ability to display discriminative facial expressions is related to their brain maturity.

## 4. Discussion

In this study, we demonstrate that discriminative facial expressions emerge at approximately 33 weeks' gestation. Older infants are more likely to display facial expressions only to the noxious stimuli, whereas younger infants are as likely to display facial expressions to either stimulation with no difference in the response duration. Overall, we demonstrate that the emergence of discriminative facial expressions coincides with the maturation of brain activity responses.

Discriminative facial expressions to tactile and noxious stimulation have previously been reported in premature infants from 26 to 31 weeks' gestation,^24^ which contrasts with the results reported here. However, this is likely due to the differences in experimental design. Although the innocuous tactile stimulus in our study was elicited by rotating the lancet by 90° and releasing the blade, Johnston et al. compared noxious heel lancing with nurse touch. The innocuous stimulus we used did not pierce the infant's skin, but was not comparable to nurse touch as it replicated all other salient aspects of the clinical heel lance procedure. Therefore, there is no suggestion from this study that positive tactile stimulation should be discouraged in the care of premature infants. Until recently, repeated tactile stimulation in extremely preterm infants was thought to be associated with hypoxemia,^29^ and a practice of minimal touch was encouraged in neonatal care,^14^ depriving infants of somatosensory stimulation that is now thought to be essential to neurodevelopment.^6,30^ Furthermore, early skin contact and positive affective tactile interventions such as kangaroo care^13^ and massage^2,22^ are thought to promote physiological stability,^4,32,39^ reduce clinical pain scores,^8,23,25^ and mitigate the attenuation of touch-evoked brain activity that has been associated with early-life pain.^30^ It is also important to note that pain experience is not dichotomous, and a limitation of this study is that pain discrimination was only considered at a single intensity with a relatively low saliency. It is plausible that if we were to consider a more intense noxious clinical procedure, such as chest drain insertion, discriminative facial expressions may be observed at an earlier gestation. Nevertheless, the demonstration of a lack of discriminative facial expressions after noxious and innocuous events in infants younger than 33 weeks' gestation highlights the need for caution when interpreting pain scores in the youngest infants. In this study, the noxious heel lance evoked a range of behavioural, neurophysiological, and autonomic responses, which were characterised across different durations lasting from milliseconds to seconds. The variation in the duration of these responses may, in part, explain why responses recorded using different measurement techniques may not necessarily be highly predictive of one another.^34,37,43^ Although a few of the younger gestation infants did display discriminative facial responses to the noxious procedure, these responses were less likely to occur than in infants approaching term gestation, and likely relate to the maturity of the individual's brain activity.

Consistent with previous research,^12,19^ we observed a developmental switch between immature delta brush responses and mature modality-specific evoked brain activity. This may be related to the disappearance of the subplate zone and the development of direct thalamocortical connections, as well as the formation of callosal and association pathways, which all occur during this developmental window.^26^ The emergence of discriminative facial expressions coincided with this developmental switch in brain activity. Further work will elucidate how the maturity of these structural pathways relates to sensory-evoked responses and the emergence of facial expression discrimination. The results of the study are limited by our choice of pain measures. Although the PIPP-R score is one of the best validated behavioural pain scores, using another behavioural measure would perhaps produce slightly different developmental timings for the emergence of discrimination. Furthermore, new measures of noxious-evoked brain activity are being developed, and time–frequency analysis of the EEG responses as well as imaging techniques, such as fMRI, could be used to further explore changes in brain activity and localise the areas from which these developmental changes in activity originate.

It is interesting to consider the results of this study in the context of an evolutionary and practical ethics perspective. Evolutionarily, it may be considered unsurprising that premature and neurologically immature infants are less likely to display discriminative behaviours. Facial expressions in infants are signals that evolve to elicit a response from caregivers.^33^ Essentially, as the evolution of these behavioural signals require a receiver,^31^ there can be no direct adaptive benefit for foetuses to use facial features to signal their experience of a noxious stimulus in utero. Thus, it is plausible that infants born very prematurely may also be less reliably able to display discriminative facial expressions after noxious and nonnoxious stimuli compared with more mature term-born infants. Facial expressions are complex behaviours, which have been observed to occur spontaneously and reflexively in utero from around 24 weeks' gestation and become increasingly complex with increasing gestation.^36^ Healthy foetuses, not subjected to aversive stimulation, show spontaneous facial expressions consistent with pain/distress, which are thought to be a sign of healthy maturation.^36^ It is possible, that these complex behaviours are essentially being practiced in utero, like other fundamental skills, such as breathing, as part of an adaptive developmental process that confers postnatal benefit to the foetus.^20^ The impact of ex utero life on the development of these behaviours is yet to be elucidated.

From an ethical perspective, a central priority for the care of preterm infants is the avoidance of unnecessary harm during treatment. Where it is uncertain whether harm may result, it is advisable to apply a precautionary principle that errs on the side of caution to prevent potential harms, even if scientific uncertainty exists about their extent.^5,40^ Our results raise fundamental questions as to how clinicians should best avoid potential harms to infants, many of which could arise from an incomplete understanding of pain. For example, failure to give infants analgesics in situations where they experience conscious pain could result in harm, but so too could providing analgesics with potential side effects in situations when they are unnecessary.

Although our data do not address the question of when infants are first capable of perceiving pain, the study does support the view that the brain's ability to discriminate noxious and innocuous tactile stimulation develops concomitantly with the emergence of differential behavioural responses to such stimuli. One possible interpretation of these results is that infants who respond facially to both noxious and innocuous tactile stimulation have a similar aversive experience in both cases. This view would suggest that pain-mitigating approaches for premature infants might be appropriate in the context of a wide range of procedures (including many not usually considered painful in older infants). At the other extreme, an alternative interpretation is that infants who are not able to discriminate between noxious and innocuous stimulation do not consciously experience pain. This view would support a much more restrictive use of analgesics in very premature newborn infants. However, regardless of whether noxious stimulation is consciously painful (which this study does not address), there is increasing evidence that painful procedures in early life are instrumentally harmful in that they can alter pain sensitivity and cognition in later life.^21,45^ Consequently, there is a critical need to limit procedures considered painful in prematurely born infants and to better manage and treat pain to mitigate long-term effects, irrespective of whether these procedures are perceptually painful.

In conclusion, we demonstrate that the emergence of facial expressions that discriminate between noxious and innocuous stimulation is concomitant with the maturation of brain responses in premature infants. This suggests that premature infants with relatively immature nervous systems display nondiscriminative facial behaviours to equally salient noxious and nonnoxious inputs, presenting challenges for the interpretation of pain and analgesia in this unique patient group. The urgent need for improved methodology to assess pain in the youngest premature infants is clear.

## Conflict of interest statement

The authors have no conflict of interest to declare.

## Appendix A. Supplemental digital content

Supplemental digital content associated with this article can be found online at http://links.lww.com/PAIN/A686.

## Supplemental video content

Video content associated with this article can be found online at http://links.lww.com/PAIN/A687.
